# Preparation and Energy Storage Properties of a Lauric acid/Octadecanol Eutectic Mixture

**DOI:** 10.1021/acsomega.1c03626

**Published:** 2021-08-31

**Authors:** Jingtao Liu, Dahua Jiang, Hua Fei, Yuzhen Xu, Zui Zeng, Weiliang Ye

**Affiliations:** Jiangxi Province Key Laboratory of Environmental Geotechnical Engineering and Hazards Control, Jiangxi University of Science and Technology, Ganzhou 341000, Jiangxi Province, China

## Abstract

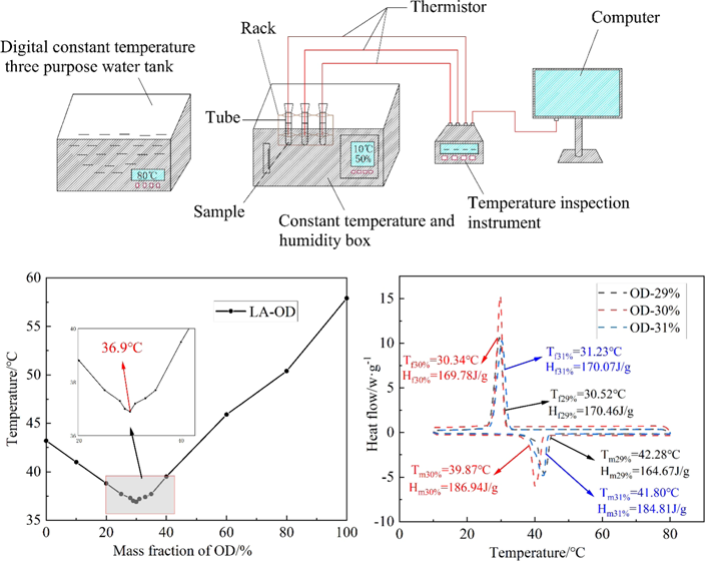

A phase change material (PCM) has the characteristics of latent heat storage, controllable phase transition temperature (PTT), and chemical stability. It can naturally regulate the ambient temperature in a certain range and reduce the load of air conditioning operation. Therefore, it plays an important role in the field of energy-saving buildings, and the PTT of PCM is one of the decisive factors. In this paper, through analyzing PCM installed in solar buildings at various regions, a binary eutectic mixture (EM) was prepared from lauric acid (LA) and octadecanol (OD) by the method of mixed melting, and the PTT and enthalpy of the EM were 39.87 °C and 186.94 J/g, respectively. The PTT, latent heat, and EM ratio were determined by theoretical calculation, the step cooling curve, and DSC. FT-IR result shows that no chemical reaction occurs among the components of composites, and the molecular forces are uniform and stable. XRD results further proves that no other phases existed in the composites. Thermal cycles (500) and the TG test show that the EM has excellent thermal stability and heat resistance, which meets the engineering application. Due to the thermodynamic properties of the EM, it can be used in thermal cooling of electronic systems, building envelopes, and thermal storage in solar buildings to obtain a good energy-saving effect.

## 1 Introduction

With the sustainable development of society, building energy consumption accounts for about 35% of the total national one, and more than 50% of it comes from air conditioning and heating.^1,2^ As special heat storage materials, a PCM controlling the ambient temperature by absorbing and releasing the heat to the surrounding is a new green material to reduce building energy consumption.^3−5^ PCM in solar buildings not only improves the heat storage of the building envelope effectively but also cuts down the load of air conditioning or heating, which is a technology worthy of great research and promotion.^6−9^

For PCM in the current building structure, the PTT is usually required to be around 20–40 °C and more rigorous in specific climatic conditions and building environments.^10−13^ The optimal PTT of PCM in buildings is related to the climate, season, and application purpose of the building, especially in solar buildings.^14,15^ Thermal regulation measures for passive solar buildings combined with a PCM wall in different seasons in North China were proposed by Chen;^16^ the PTT of the PCM is about 20 °C, and it is feasible at other similar climate areas. An ideal model of thermal PCM storage floor heated by an electric power system was established by Lin,^17^ operating from 23:00 to 7:00 at night to simulate the application effect of the system in Beijing, Shanghai, Dalian, and Harbin, and the floor with PCM maintaining the indoor temperature in the range of 16–25 °C in winter, which basically meets the thermal comfort except for Harbin. Peippo et al.^18^ studied the thermal performance of passive solar houses in different areas of the US and concluded that the PTT of PCM should be 1–3 °C higher than the indoor average temperature. Yang et al.^19^ found that the PTT range of the roof structure is 33–35 °C under typical daytime weather in summer in Wuhan area. Yu et al.^20^ used the PCM on the roof with the PTT of 37 °C under conditions of the summer climate, and it reduced the indoor heat transfer by 46.71% compared with the reference roof. Wang^21^ studied the energy-saving effect of solar ventilation and the phase change heat storage wall in buildings in South China, in the daytime of transition season, and the maximum temperature of the outer surface wall is about 40 °C, therefore, the higher PTT of PCM should be considered. Chen et al.^22^ introduced a new type of phase change energy storage wallboard, and the energy-saving rate in the heating season can reach 17% or even higher. Similarly, in the field of building thermal storage and thermal cooling of electronic systems, many scholars have done a lot of research.^23,24^ A PCM-air heat energy exchanger was designed to collect the available solar energy and provide thermal comfort, and the PTT of such PCM is determined to change around 37 °C.^25,26^ A paraffin-based PCM with a melting point in range of 38–43 °C is used in a PV/PCM system for cooling, which increased the electrical efficiency and reduced energy consumption in the hot areas of United Arab Emirates.^27^ Maccarini et al.^28^ found that replacing the cooling system with a PCM-based heat exchanger saves about 60% of energy consumption in a thermal plant.

In the application of PCM, appropriate thermodynamic properties are very important. Organic PCM is the most common heat storage material in thermal energy storage systems, and it is often used in the field of building envelopes, solar heating and cooling of buildings, etc. because of the excellent thermodynamic and kinetic properties.^29−31^ Lauric acid (LA) is a kind of saturated fatty acid organic PCM, which has the advantages of high latent heat, good chemical and thermal stability, and almost no supercooling and pollution.^32,33^ Due to its suitable PTT, it was often synthesized with other PCMs and applied in engineering. Many scholars used LA to prepare binary shape-stabilized CPCM with excellent thermal properties for building solar energy utilization, cold storage, and air conditioning.^34−36^ For example, He et al.^37^ prepared a binary phase change mortar by adding LA and MA , which can play a great role in temperature control and delay the temperature change. In addition, a thermal storage wallboard was prepared from CA–LA EM and gypsum board, and it reduces the heating load of air conditioning in the house.^38,39^ Octadecanol (OD) has high thermal conductivity, a large energy storage density, and stable phase change performance and belongs to a fatty alcohol organic PCM, and the CPCM prepared by adding OD can display excellent thermal properties.^40−42^ Wang et al.^43^ developed a CPCM by adding OD that has good thermal conductivity. In another study, a CPCM with high shape stability and thermal stability was prepared from OD with thermal energy storage.^44^ Fatty acids and fatty alcohols can form a binary system at the lowest melting point, and no new material phase appears, and the change of PTT expands their application region of the materials.^45−47^

In this paper, a binary EM of LA-OD was prepared by mixed melting. The microtopography, phase characteristics, thermal performance, and stability of the EM were tested and determined.

## 2 Results and Discussion

### 2.1 Proportion of Theoretical Calculation

First, the minimum of crystallization temperature and the optimum ratio of binary EM are calculated as follows:

Using Schroder eqs 1–4, the theoretical phase diagram of the binary eutectic system can be obtained and the eutectic point can be determined.^48^
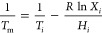
1

The molecular weight, melting point temperature, and latent heat of LA and OD are substituted in the formula, respectively
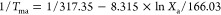
2
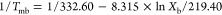
3
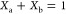
4

**Figure 1:**
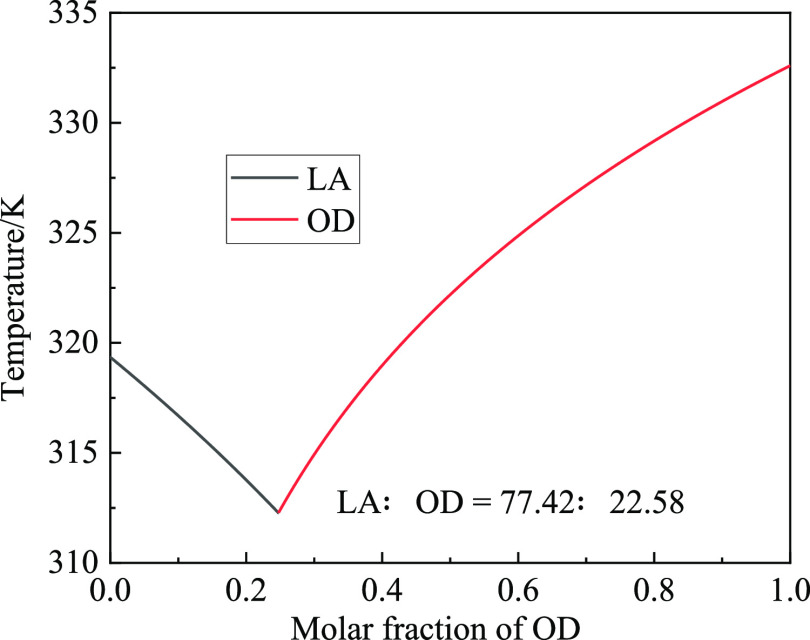
Theoretical phase diagram with different molar ratios of OD.

It can be concluded that the molar ratio of LA-OD EM is 77.42:22.58, the PTT is 37.88 °C, and the predicted molar ratio phase diagram is shown in Figure 1.

According to formulas 5 and 6, the molar ratio is converted to the mass ratio, *M*_LA_/*M*_OD_ = 71.74:28.26.
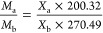
5
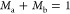
6

### 2.2 Theoretical Calculation of Latent Heat

The theoretical transformation latent heat of N-element EM is calculated as follows
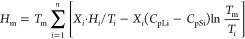
7

The above formula 7 can be simplified to formula8.^49,50^
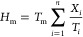
8

Finally, according to the formula, the latent heat of LA-OD is 172.31 J/g.

### 2.3 Experimental Proportion Determination of LA-OD Binary EM

**Figure 2:**
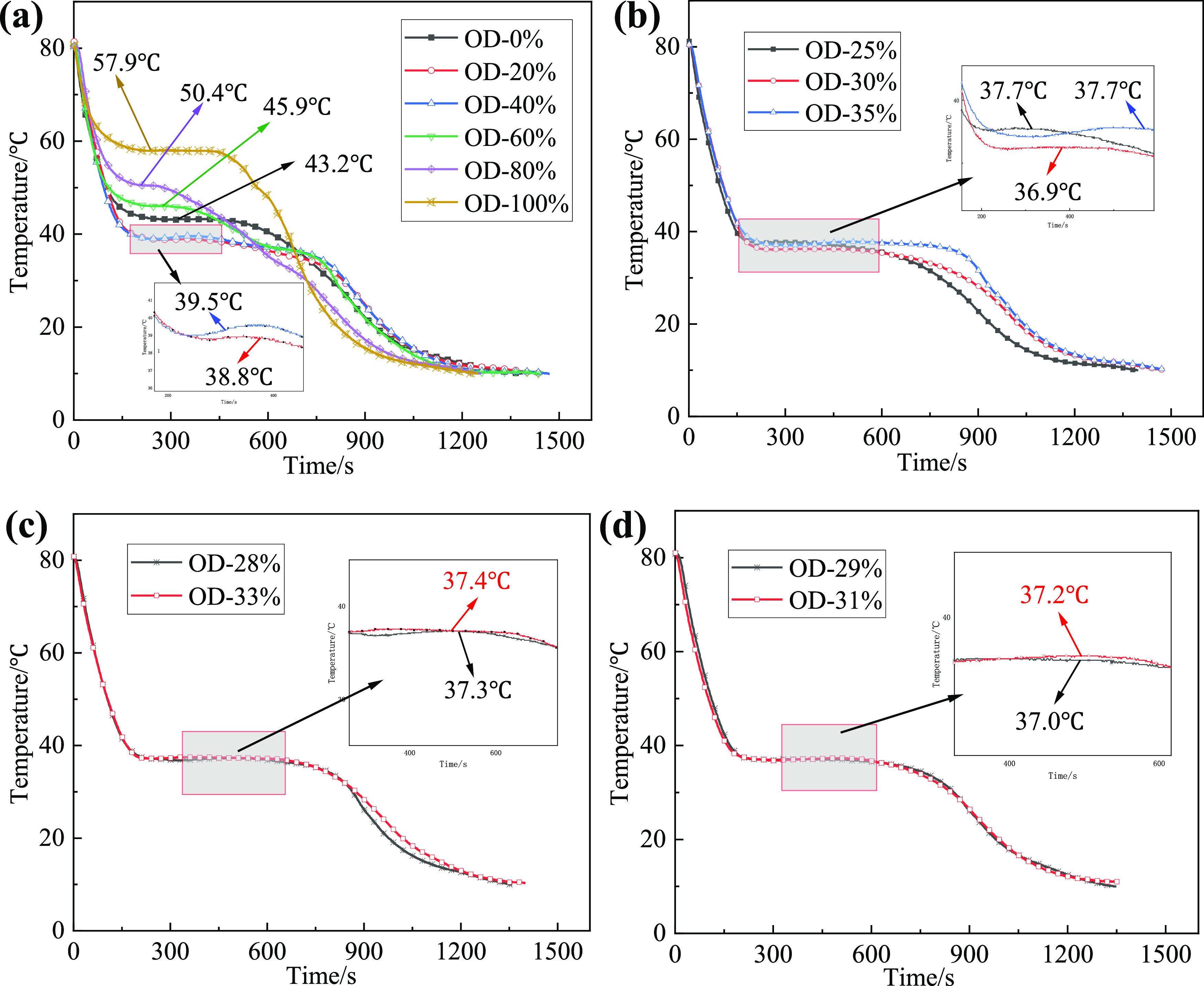
Step cooling curves of different OD mass fractions: (a) 0–100 wt % of OD; (b) 25, 30, and 35 wt % of OD; (c) 28 and 33 wt % of OD; and (d) 29 and 31 wt % of OD.

The lowest melting point and the best mass ratio of binary EM are determined by dichotomy gradually. It shows the step cooling curve of the LA-OD binary system with OD mass fractions of 0, 20, 40, 60, 80, and 100% in Figure 2a. The results show that phase transformation occurs in LA-OD composites during the cooling process, and the crystallization temperatures of LA and OD are 43.2 and 57.9 °C, respectively. When the mass fractions of OD are 20 and 40%, the crystallization temperature of the binary eutectic mixture is 39.5 and 38.8 °C, respectively. Therefore, the mass fraction of OD at the lowest melting point ranges from 20 to 40%, and the phase diagrams of the composite system with step cooling curves are drawn in Figure 2b, in which the mass fractions of OD are 25, 30, and 35 wt % and PTTs are 37.7, 36.9, and 37.7 °C, respectively. In Figure 2c, the mass fractions of OD are 28 and 33% and PTTs are 37.3 and 37.4 °C, respectively. In Figure 2d, the mass fractions of OD are 29 and 31% and the PTTs are 37.0 and 37.2 °C, respectively.

**Figure 3:**
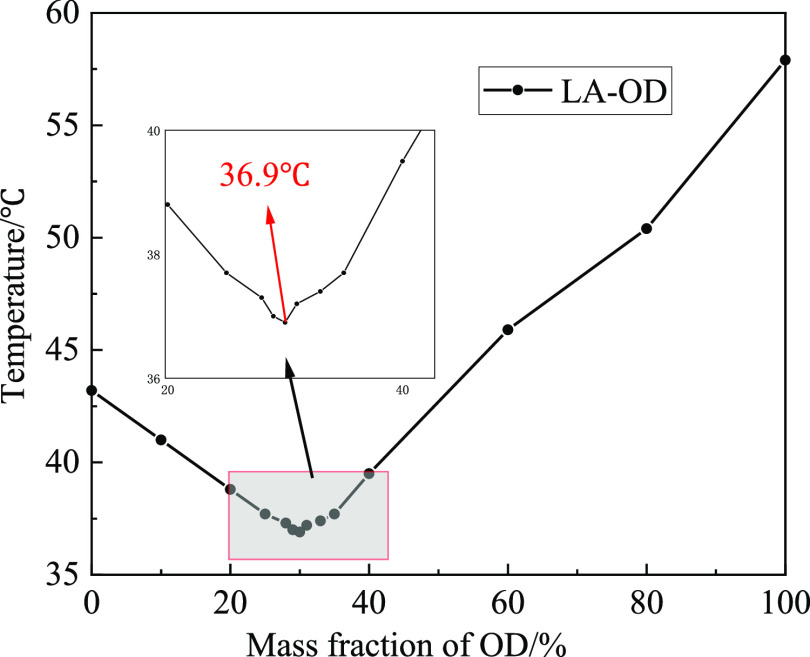
Experimental phase diagram of the LA-OD binary system.

As seen from the above step cooling curves of binary EM, the PTT diagram of the LA-OD eutectic system changing with the ratio is shown in Figure 3. It can be determined that the mass ratio of LA and OD is 70:30, and the lowest PTT of binary EM is 36.9 °C.

### 2.4 Characterization of Thermal Performance

**Figure 4:**
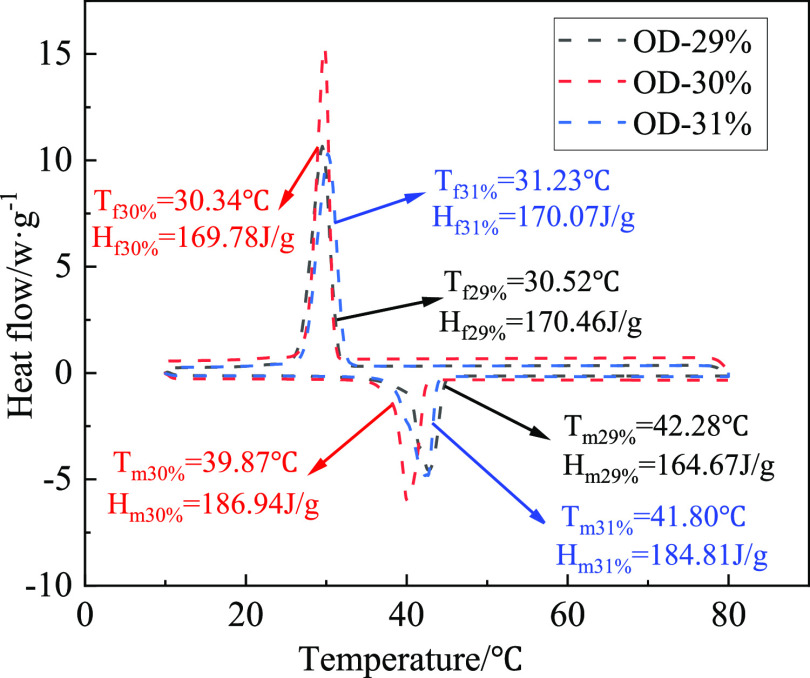
DSC curve of the LA-OD binary system.

The LA-OD EM samples were tested by DSC with mass fractions of 29, 30, and 31%. Under the nitrogen atmosphere with a flow rate of 50 mL/min, the sample was cooled to 10 °C and kept for 5 min by liquid nitrogen, then heated to 80 °C and kept for 5 min, and then cooled to 10 °C. The rising and cooling rate was set at 5 °C/min. The results of the temperature heat flux curve are shown in Figure 4. *T*_f_ and *H*_f_ are the solidification temperature and latent heat of LA-OD, respectively, while *T*_m_ and *H*_m_ are the melting temperature and latent heat. The PTTs of LA-OD with OD mass fractions of 29, 30, and 31% are 42.28, 39.87, and 41.80 °C, respectively. The latent heat is 164.67, 186.94, and 184.81 J/g, respectively. The change rule of the melting process is basically consistent with the solidification process. The PTT of LA-OD is the lowest when the mass fraction of OD becomes 30%. Therefore, 39.87 °C and 186.94 J/g are the corresponding PTT and latent heat of the LA-OD with the best ratio.

### 2.5 Analysis of the Molecular Structure

**Figure 5:**
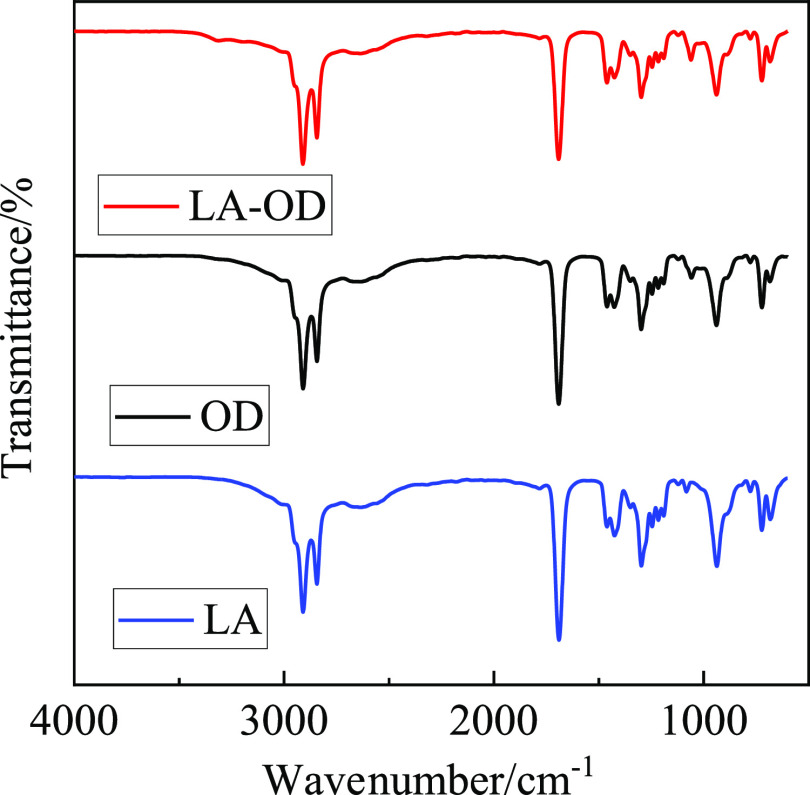
FT-IR curves.

The LA, OD, and LA-OD EM were tested by FT-IR. The test of frequency is 4000–400 cm^–1^, the resolution is 4 cm^–1^, and the sample and background scanning times are 32. The result of the infrared spectrum is shown in Figure 5. The results show that there is a C–H asymmetric stretching vibration absorption peak at 2909 cm^–1^ in the FT-IR curve of LA, the characteristic absorption peak caused by the stretching vibrations of C=O appears at 1690 cm^–1^, the bending vibration absorption peak of −OH appears at 1426 cm^–1^, and the characteristic absorption peaks at 1297, 937, and 723 cm^–1^ are caused by the stretching vibrations of C–O, out-of-plane bending vibrations of −OH, and in-plane bending vibrations of −OH, respectively, caused by bending vibrations. The FT-IR spectra of OD at 2909 cm^–1^ is produced by the antisymmetric stretching vibrations of −CH_3_, the stretching vibrations of C=O at 1691 cm^–1^, the bending vibrations of −OH at 1427 cm^–1^, and the stretching vibrations of C–O, out-plane bending vibrations of −OH, and in-plane bending vibrations of −OH at 1298, 939, and 722 cm^–1^, respectively, caused by bending vibrations. The FT-IR curve of LA-OD is basically the same as that of LA-OD, and there is no new characteristic absorption peak, though the position and transmittance of the characteristic absorption peak change slightly. Therefore, it shows that LA and OD are the uniform and stable fusion due to intermolecular forces, no chemical reaction occurs, and no new substance grows.

### 2.6 Analysis of the Crystal Structure and Composition

**Figure 6:**
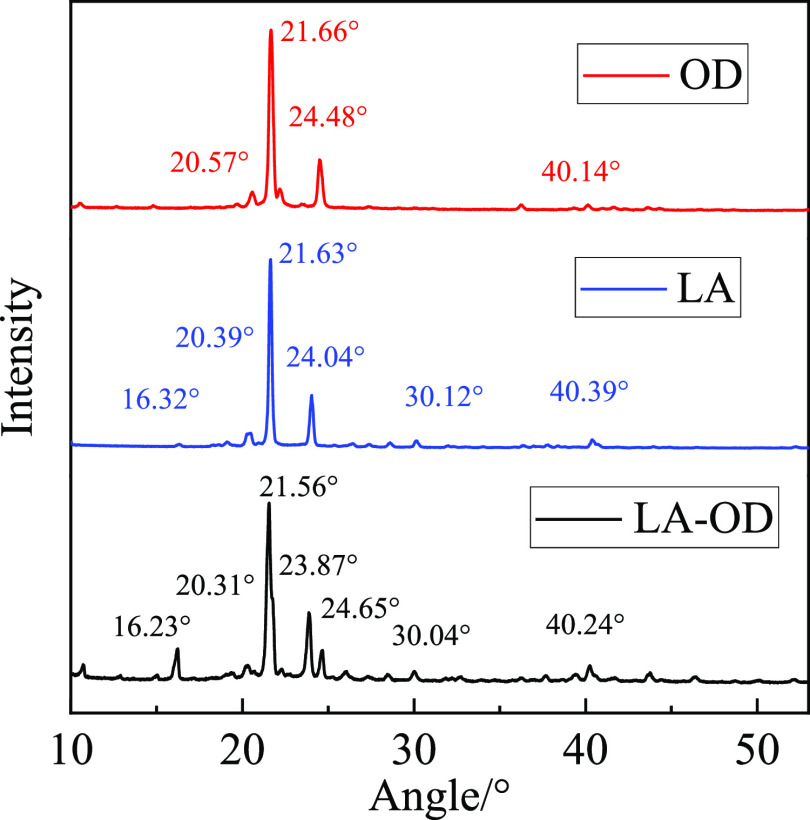
XRD curves.

XRD patterns of LA, OD, and LA-OD EM are shown in Figure 6. The scanning angle is 10–65°, and the scanning rate is 5°/min. The angles of the obvious characteristic peaks of LA are 16.32, 20.39, 21.63, 20.04, 30.12, and 40.39°. The angles of the obvious characteristic peaks of OD are 20.57, 21.66, 24.48, and 40.14°. The angles of the characteristic peaks of the LA-OD EM system are 16.23, 20.31, 21.56, 23.87, 24.65, 30.04, and 40.24°. The main phases corresponding to LA and OD are reflected in the binary eutectic system, and their angles are basically consistent, which further proves that the composite PCM of LA-OD is a uniform and stable combination without the new phase.

### 2.7 Comparative Analysis of Heat Storage and the Release Experiment

**Figure 7:**
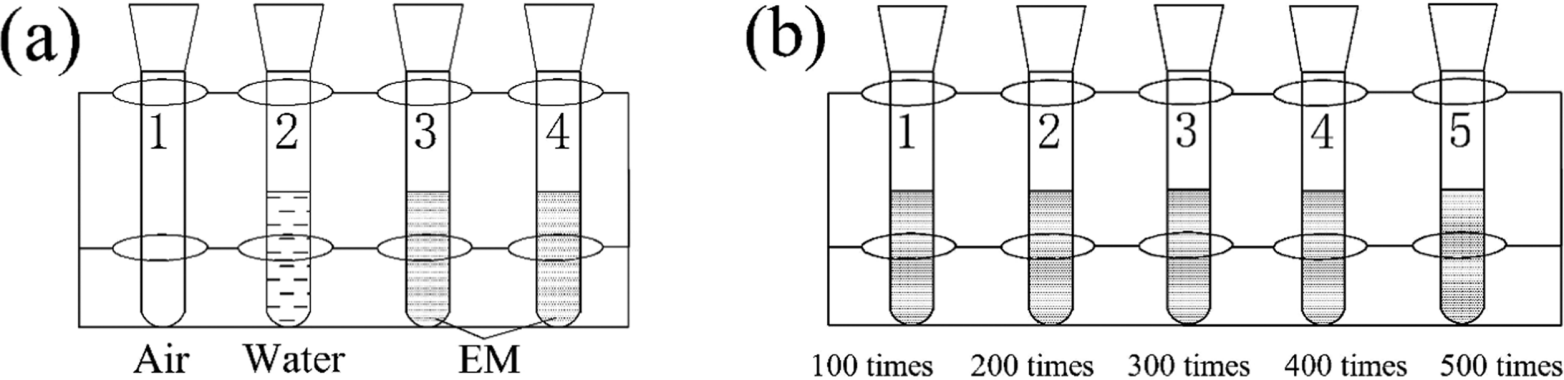
Test tube style chart: (a) heat storage and release test and (b) thermal cycling test.

**Figure 8:**
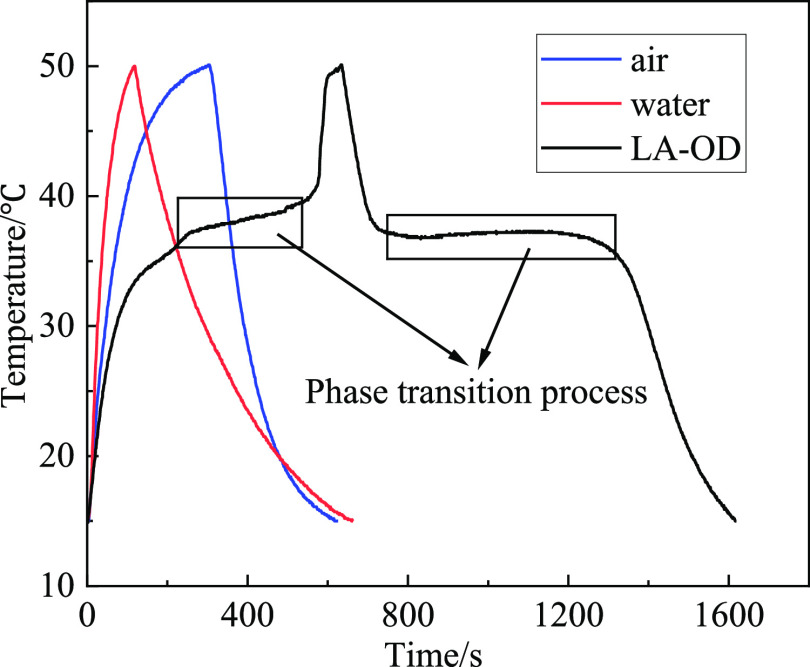
Heat storage and release curves.

**Figure 9:**
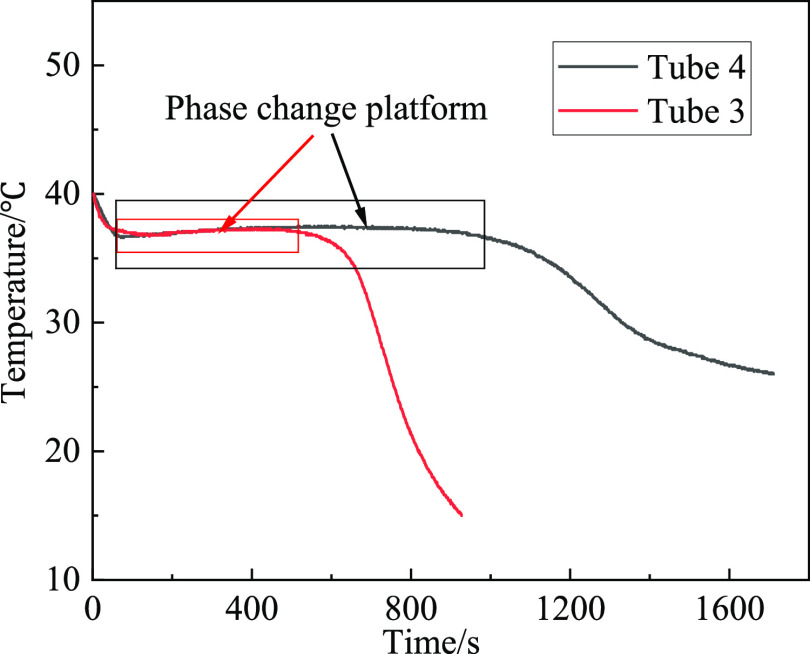
Cooling curves with time change.

The temperature changes in a complete endothermic and exothermic cycle of air, water, and LA-OD EM under the set environment and LA-OD under natural conditions were measured. Tube 1 was filled with air, tube 2 was pure water, and tubes 3 and 4 had equal amounts of LA-OD, as shown in Figure 7a. First, tubes 1, 2, and 3 were put in a constant temperature and humidity incubator, when the temperature can be maintained at 15 °C, they were taken out immediately and heated to 50 °C by water. At the same time, the temperature inspection instrument connected with the thermal resistance wire began to record data. When the temperature increases to 50 °C, the tubes were taken out quickly and put into 15 °C constant temperature and a humidity incubator. When the temperature drops to 15 °C, a cycle is completed, and the data record is shown in Figure 8. The test tube 4 was placed in the natural environment cooling from 40 to 25 °C, and it simulates the temperature change of the EM under the actual condition. The cooling curves of tube 3 and tube 4 are shown in Figure 9.

As seen in Figure 8, for the total heating and cooling cycle, the time of tube 1 and tube 2 is about 650 s, and that of tube 3 is about 1620 s, which is 2.5 times as much as tube 1 and tube 2, and the difference of temperature rising and fall curves between tube 1 and tube 2 is mainly due to the greater thermal conductivity of water than air. The LA-OD in tube 3 undergoes six processes: absorption of solid sensible heat, absorption of latent heat, absorption of liquid sensible heat, releasing of liquid sensible heat, and releasing of latent heat and solid sensible heat. Compared with the materials in tubes 1 and 2, the EM in tube 3 can absorb and store most of the heat during heating, and it releases heat slowly during cooling, and time delays when the temperature is the highest and the lowest. In the process of melting and heat absorption of the EM, it lasts about 240 and 400s on the temperature platform, respectively. This indicates that the EM has excellent performance of heat storage and temperature controlling.

As shown in Figure 9, it takes 930 s for the EM temperature in tube 3 to fall from 40 to 25 °C, and the duration of the phase transition platform is around 450 s. It takes 1710 s for the EM temperature in tube 4 to fall from 40 to 25 °C, and the duration of the phase transition platform is around 920 s. The results indicate that the EM can last a longer time in the environment with a smaller PTT difference, and the material has a better ability of absorbing and storing latent heat under the conditions of normal temperature.

### 2.8 Analysis of Thermal and Chemical Stability

**Figure 10:**
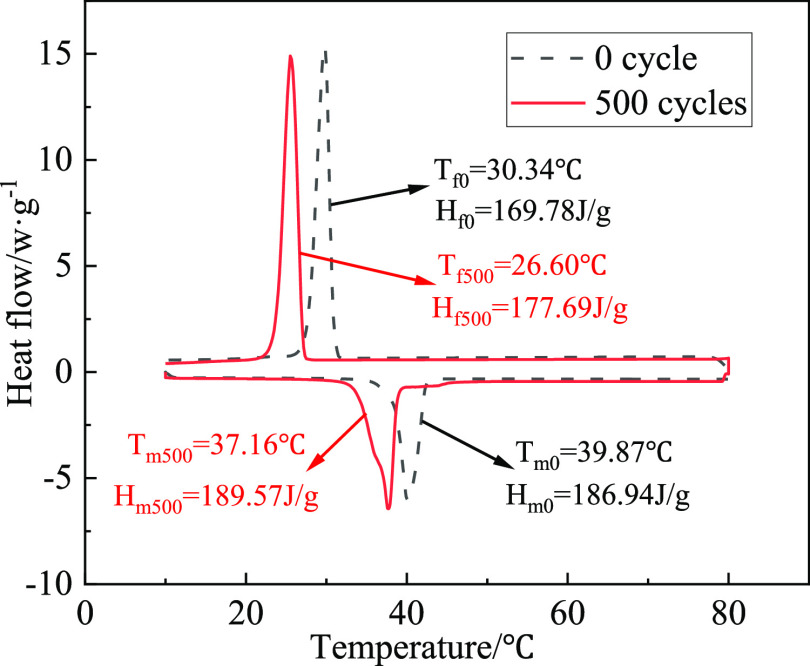
DSC curves of the LA-OD binary system after 500 cycles.

**Figure 11:**
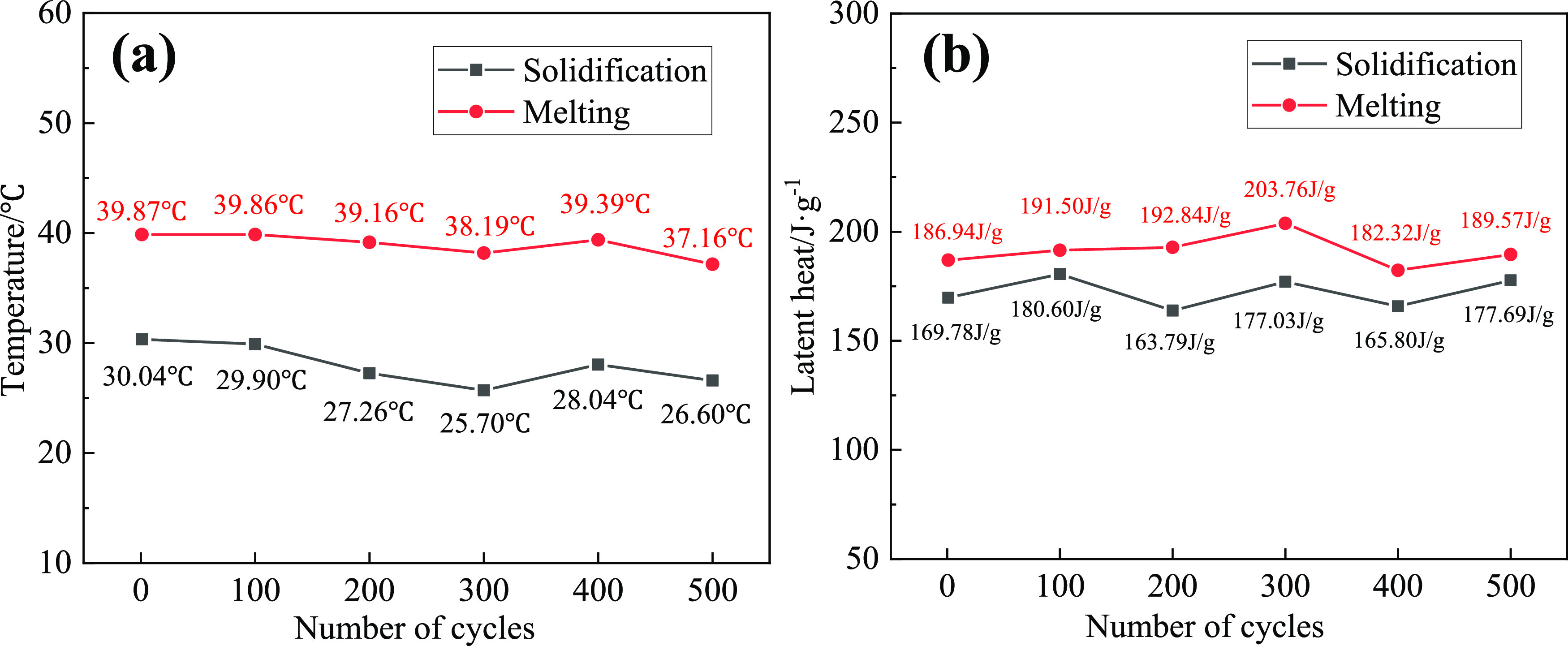
Changes of temperature and latent heat: (a) temperature and (b) latent heat.

Divide LA-OD EM into five equal parts and put into the test tubes. The five groups of samples were labeled as 1, 2, 3, 4, and 5, standing for the corresponding 100, 200, 300, 400, and 500 times of the cooling and heating accelerated process, as shown in Figure 7b. DSC curves of two groups of samples in the 0 time and 500 times are expressed in Figure 10. Changes of the PTT and the latent heat after a thermal circulation of 0 time, 100 times, 200 times, 300 times, 400 times, and 500 times are described in Figure 11a,b.

**Figure 12:**
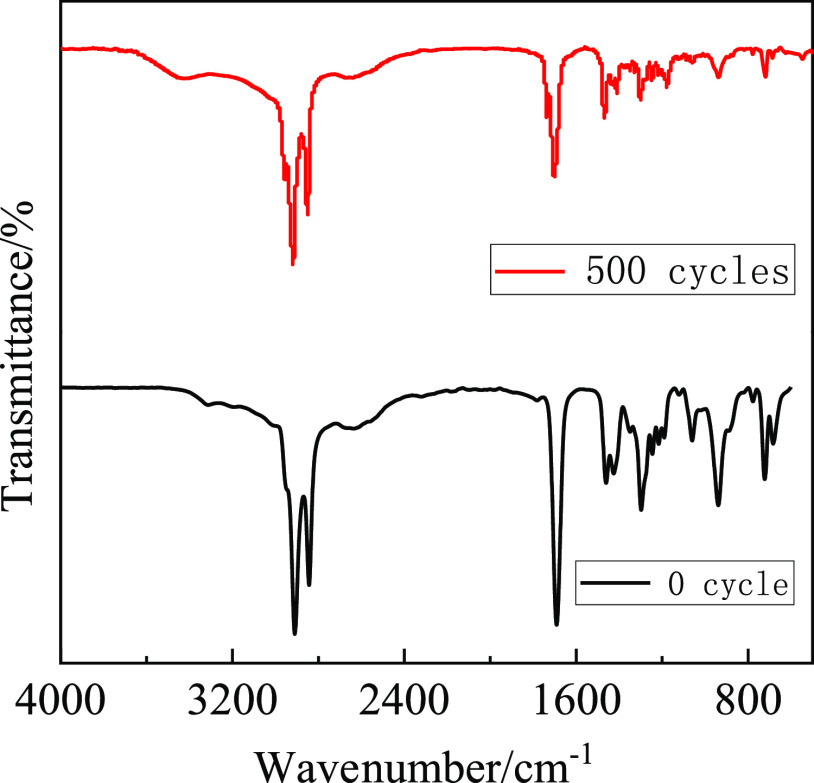
FT-IR curve of LA-OD after 500 cycles.

As shown in Figure 10, the DSC curve of the EM has almost no change after 500 times of the heating and cooling process, and the thermal property of the material remains stable. The melting and solidification temperatures after 500 cycles were 37.16 °C and 26.60 °C, respectively, and the latent heat of melting and solidification were 189.57 and 177.69 J/g, respectively. Comparing the phase transformation performance of LA-OD before and after cold and hot cycles, it can be found that the melting and solidification temperatures are reduced by 2.71 and 3.44 °C, respectively, and the melting and solidification latent heat are increased by about 1.4 and 4.6%, respectively. As shown in Figure 11a,b, the PTTs of the EM are 39.87, 39.86, 39.16, 38.19, 39.39, and 37.16 °C, and the latent heat values are 186.94, 191.50, 192.84, 203.76, 182.32, and 189.57 J/g, respectively. The solidification temperatures of the EM are 30.04, 29.90, 27.26, 25.70, 28.04, and 26.60 °C, and solidification latent heat values are 169.78, 180.60, 163.79, 177.03, 165.80, and 177.69 J/g, respectively. There are some differences in temperature and latent heat values, which may be caused by the purity of materials, the accuracy of instruments, and the deviation of operation. During the melting and solidification process of the EM, the change trend of latent heat and PTT is basically the same. Compared with the 0 cycle, the PTT descends less than 1 °C every 100 times during the 300 thermal cycles, and the PTT of the EM rises slightly while 400 cycles are completed, which shows that the material is basically stable at this time. After 500 cycles, the PTT of the EM descended about 2.7 °C, and the change of the PTT of the EM is not obvious. The latent heat of EM did not descend but increased 2.63 J/g. It is speculated that the combination of molecules becomes more compact after the cooling and heating process. The result shows that the EM has excellent phase change reversibility, high latent heat and strong heat storage, and heat release ability. The EM was tested by FT-IR after 500 cycles. As shown in Figure 12, there is no new characteristic absorption peak, and its characteristic peaks are basically consistent with that of the 0 cycle. Therefore, the EM is considered to have good thermal and chemical stability.

### 2.9 Analysis of Heat Resistance

**Figure 13:**
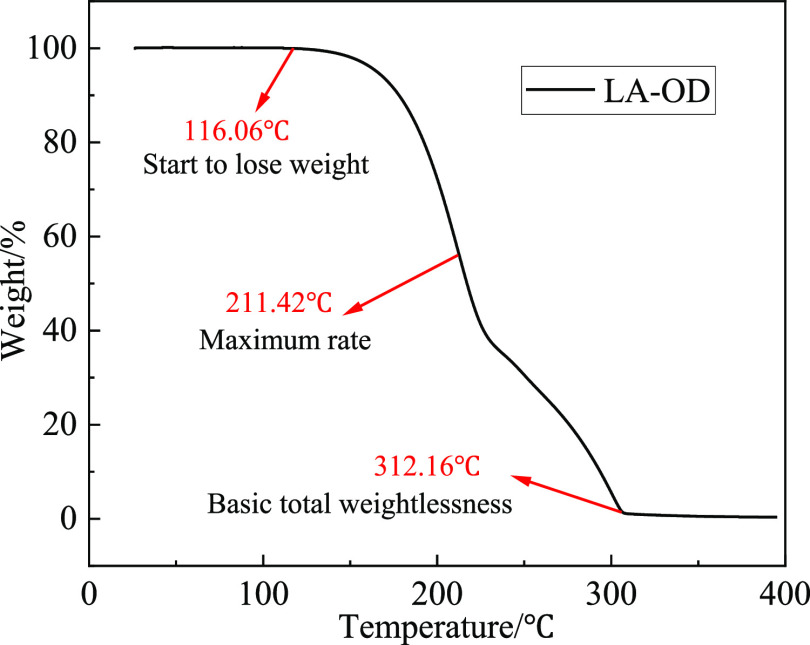
TG curve of LA-OD.

Operating for 500 cycles, LA-OD was subjected to the TG test, and the temperature increased from room temperature to 400 °C at a heating rate of 10 °C/min in a nitrogen atmosphere, and the nitrogen atmosphere with the flow rate of 100ml/min. As shown in Figure 13, the initial weight loss temperature of EM is about 116.06 °C, and the main weight loss range is 116.06–312.16 °C. When the temperature reaches 116.06 °C, the EM begins to decompose. At first, with the increase of the temperature, the chemical bonds inside the LA begin to break and decompose into water and carbon dioxide. Then, as the temperature rises further, OD began to decompose, and due to the simultaneous decomposition of LA and OD, the weight loss rate reaches the fastest at 211.42 °C. When LA is basically decomposed and only OD is left, it comes to the second stage and the decomposition speed decreases, at this time, the temperature is about 232 °C. When the temperature reaches 312.16 °C, the weight loss rate exceeds 99%, which indicates that the EM will completely decompose at high temperature. Such a high temperature could not appear in the normal building environment. Therefore, the EM can meet the application requirements in the decomposition temperature range.

## 3 Conclusions

A binary EM was prepared by the melt mixing method with a strong capacity of heat storage and release, which was successfully combined by the good interaction of the molecules. The preparation process of the EM is concise, and the PTT of EM reduces that of a single one, which broadens the application scope, and a new choice is provided for building energy-saving materials. The PTT, enthalpy, and optimum proportion of the EM a measured to be 39.87 °C, 186.94 J/g, and 70:30 for LA-OD, respectively. Compared with the DSC test, the error of theoretical calculation is about 1.99 °C and 1.74% in PTT and the mass ratio, respectively; the error of the step cooling curve method is about 2.88 °C in PTT, and the mass ratio of experimental results is basically consistent with it. It shows that the minimum eutectic calculation theory and step cooling curve method have important guidance for the configuration of the organic binary eutectic system. Tests of thermal storage and release improved an excellent effect for the temperature regulation in experiments. It can be concluded that the material has good thermal and chemical reliability by the thermal cycling test. Moreover, the TG analysis after 500 thermal cycling shows that the EM has an excellent heat resistance. In view of the PPT and properties of the EM, it can be applied in thermal cooling of electronic systems, building envelopes, and thermal energy storage in solar buildings.

## 4 Materials and Methods

### 4.1 Materials

Lauric acid (LA, CP, 44.20 °C of PTT, 200.32 of molecular weight) and octadecanol (OD, CP, 59.45 °C of PTT, 270.49 of molecular weight) were supplied by Guoxue Group Chemical Reagent Co., Ltd (Shanghai, China).

### 4.2 Preparation of (LA + OD)

The EM was synthesized by way of mixed melting. The minimum of the crystallization temperature, latent heat, and mixing mass ratio of LA and OD were calculated by the formula developed. LA and OD were mixed in a mass ratio of 70:30 in a glass beaker at 80 °C for 30 min to obtain binary eutectic.

### 4.3 Analysis Methods

**Figure 14:**
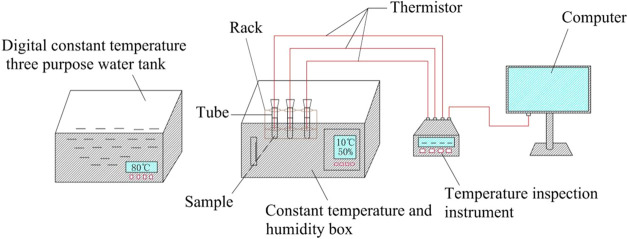
Experimental setup.

The optimum mass ratio of the PTT was verified by the step cooling curve, and the related experimental setup is shown in Figure 14. The functional groups of the EM were analyzed by FT-IR. The crystal structure of the EM was determined by XRD, and the enthalpy and thermal stability of the EM were measured by DSC and an accelerated cooling and heating device, respectively. The energy-saving effect was decided by comparative analysis of heat storage and release. The heat resistance was tested by TG.

## Nomenclature

*T*_m_: phase transition melting point (K)
*T*_*i*_: melting temperature of the *i* component (K)
*H*_m_: melting latent heat of the eutectic mixture (kJ/mol)
*H*_*i*_: melting latent heat of the *i* component (kJ/mol)
*R*: gas constant of 8.315 kJ/(mol·K)
*X*_*i*_: mole fraction of the *i* component
*C*_PL*i*_: specific heat of the *i* component at constant pressure in the liquid state
*C*_PS*i*_: specific heat of the *i* component at constant pressure in the solid state
*Mi*: mass fraction of the *i* component

## Abbreviations Used

PTT: phase transition temperature (°C)
PCM: phase change material
CPCM: composite phase change material
EM: eutectic mixture
LA: lauric acid
OD: octadecanol
CA: capric acid
MA: myristic acid
PV: photovoltaics
DSC: differential scanning calorimetry
FT-IR: Fourier transform infrared spectrometer
XRD: X-ray powder diffractometer
TG: thermogravimetric analyzer
